# Decreased mean platelet volume predicts poor prognosis in patients with pancreatic cancer

**DOI:** 10.1186/s12893-020-00976-5

**Published:** 2021-01-06

**Authors:** Takuki Yagyu, Hiroaki Saito, Teruhisa Sakamoto, Ei Uchinaka, Masaki Morimoto, Takehiko Hanaki, Joji Watanabe, Tomoyuki Matsunaga, Manabu Yamamoto, Naruo Tokuyasu, Soichiro Honjo, Yoshiyuki Fujiwara

**Affiliations:** 1 Division of Gastrointestinal and Pediatric Surgery, Department of Surgery, School of Medicine, Faculty of Medicine, Tottori University, Tottori, Japan; 2 Department of Surgery, Japanese Red Cross Tottori Hospital, 117 Shotoku-cho, Tottori, 680-8517 Japan

**Keywords:** Carbohydrate antigen 19-9, Mean platelet volume, Pancreatic cancer, Prognosis

## Abstract

**Background:**

Decreased mean platelet volume (MPV) predicts poor prognosis in some cancers. However, its significance as a prognostic indicator in pancreatic cancer (PC) remains unclear.

**Methods:**

A total of 91 PC patients who underwent pancreatectomy were included in this study. MPV and serum carbohydrate antigen 19-9 (CA19-9) were measured within 1 week before surgery.

**Results:**

We divided patients into MPV^high^ (≥ 8.65; n = 40), MPV^low^ (< 8.65; n = 51), CA19-9^high^ (≥ 66.3; n = 47), and CA19-9^low^ (< 66.3; n = 44) groups based on the optimal cut-off values determined from receiver operating characteristic curve analysis. The 5-year overall survival (OS) rates were significantly lower in the MPV^low^ than in the MPV^high^ group (16.9% and 56.3%, respectively; *P* = 0.0038), and the 5-year disease-specific survival (DSS) rates in the MPV^low^ group and MPV^high^ group were 20.5% and 62.2%, respectively (*P* = 0.0031). Multivariate analysis identified MPV as an independent prognostic indicator for both OS and DSS. The patients were then divided into groups A (MPV^high^ and CA19-9^low^), B (MPV^high^ and CA19-9^high^), C (MPV^low^ and CA19-9^low^), and D (MPV^low^ and CA19-9^high^), with 5-year OS rates of 73.2%, 40.4%, 25.8%, and 10.3%, respectively (*P* = 0.0002), and 5-year DSS rates of 80.8%, 44.9%, 27.3%, and 16.4%, respectively (*P* = 0.0003).

**Conclusions:**

Classification based on MPV and CA19-9 might be useful for predicting long-term outcomes in patients with PC.

## Background

Predicting the prognosis of cancer patients is important for determining treatment strategies and enabling informed consent to be obtained from patients and their families for treatment of various cancer types. Many prognostic indicators have been identified to date; however, the application of these indicators is difficult because of the expense and complexity of their measurements in routine clinical settings. There is thus a need to identify less invasive and more convenient prognostic indicators. Data obtained from routine blood examinations meets these criteria. A complete blood count (CBC) is an easy and convenient blood test that can be performed in most clinics. The neutrophil to lymphocyte ratio is an indicator that can be evaluated from CBC data, and which is known as a useful prognostic indicator in some cancers [1, 2].

Platelets play a pivotal role in cancer metastasis and progression [3]. Because platelet-related parameters can be obtained from a CBC, their usefulness as prognostic indicators has been determined in cancer patients, and a close correlation between an increase in peripheral platelets and a poor prognosis has been reported in various cancer types [4–6]. Mean platelet volume (MPV) is a commonly used index of platelet size that has recently attracted attention as a novel prognostic indicator. Indeed, some studies have reported that decreased MPV predicted a poor prognosis in patients with invasive bladder cancer [7] and non-small-cell lung cancer [8, 9].

Pancreatic cancer (PC) is one of the most aggressive tumors with a 5-year overall survival (OS) rate of less than 5% worldwide [10, 11]. The clinical usage of carbohydrate antigen 19-9 (CA19-9) as a marker in PC patients is recommended by the National Comprehensive Cancer Network guidelines. However, approximately 10% of Japanese patients have a Lewis blood group-negative phenotype and show false-negative findings [12, 13], indicating that CA19-9 alone is not enough to precisely predict the prognosis of PC patients. Other serum markers are therefore required to predict the prognosis of PC patients more precisely than using CA19-9 alone. We conducted this study to assess the prognostic value of MPV in patients with PC.

## Methods

This study included 91 patients with pancreatic ductal adenocarcinoma who underwent pancreatoduodenectomy, distal pancreatomy, or total pancreatomy at our institution between January 2008 and December 2017. The eighth edition of the Union for International Cancer Control (UICC) Tumor, Node, Metastasis (TNM) staging system was used to determine the patients’ clinicopathological characteristics [14]. The seventh edition of the General Rules for the Study of Pancreatic Cancer by the Japan Pancreas Society was used to evaluate lymphatic invasion, venous invasion, and perineural invasion [15].

Thirteen patients (14.3%) were treated with neoadjuvant chemotherapy (gemcitabine and S-1, n = 10; S-1, n = 2; gemcitabine and Nab-paclitaxel, n = 1) and 52 patients (57.1%) were treated with postoperative adjuvant chemotherapy (S-1, n = 39; gemcitabine, n = 13). The patients visited our outpatient clinic periodically and underwent blood tests and diagnostic imaging, including computed tomography and magnetic resonance imaging.

Preoperative data including serum CA19-9 level, total platelet count, MPV, and platelet distribution width (PDW) were measured within 1 week before surgery.

This study was approved by Certified Review Board, Tottori University Hospital and the requirement for informed consent was waived.

### Statistical analyses

Clinicopathological characteristics were compared using χ^2^ tests and the correlation between MPV and serum CA19-9 level was determined by Spearman’s rank correlation coefficient. Receiver operating characteristic (ROC) curve analysis was used to determine the Youden index and area under the curve (AUC). Survival curves were generated according to the Kaplan–Meier method and differences were examined using log-rank tests. Multivariate analyses were performed using Cox’s proportional hazards model. *P* < 0.05 was considered significant. Statistical analyses were carried out using Stat View (Abacus Concepts, Inc., Berkeley, CA, USA) and GraphPad Prism (GraphPad Software, Inc., La Jolla, CA, USA) software.

## Results

**Fig. 1:**
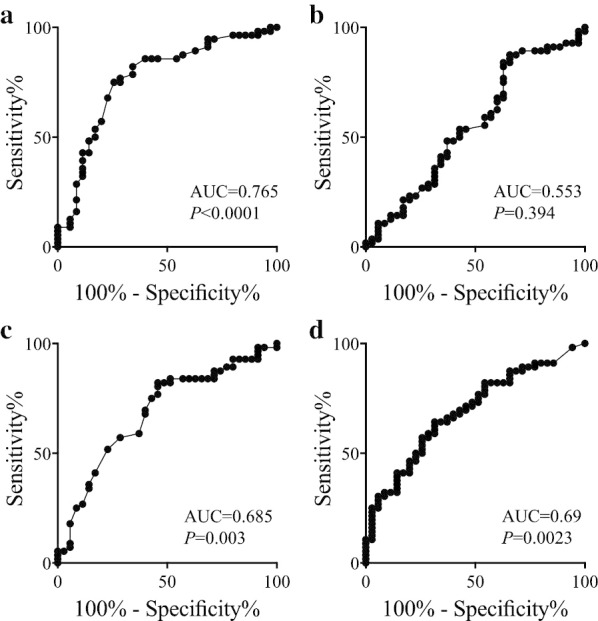
Receiver operating characteristic curves for overall survival. **a** MPV, **b** platelet count, **c** PDW, and **d** CA19-9

We first compared the AUC values of the ROC curves for platelet-related indicators (platelet count, MPV, PDW) and CA19-9 in relation to OS. The AUC was highest for MPV among the four indicators (Fig. 1), indicating that MPV was the most useful prognostic indicator. Patients were then divided into MPV^high^ (≥ 8.65; n = 40) and MPV^low^ (< 8.65; n = 51) groups based on the optimal cut-off value from ROC curve analysis. Regarding the correlations between MPV and clinicopathological characteristics, the frequency of ly2/3 was significantly higher in patients with MPV^low^ than in those with MPV^high^ (*P* = 0.005), while the frequency of v2/3 was significantly lower in patients with MPV^low^ than in those with MPV^high^ (*P* = 0.011). Although chemotherapy had the potential to affect MPV, there was no significant relationship between neoadjuvant chemotherapy and MPV (*P* = 0.77; Table 1). Furthermore, there was no difference in the usage of antiplatelet drugs between the MPV^low^ and MPV^high^ groups (*P* = 0.77).

**Table 1:** Correlations between the mean platelet volume (MPV) and patients’ clinicopathological characteristics

	MPV^High^ (n = 40) n (%)	MPV^Low^ (n = 51) n (%)	P value
Age			
< 75 Years	21 (52.5%)	32 (62.7%)	0.39
≥ 75 Years	19 (47.5%)	19 (37.3%)	
Sex			
Male	22 (55.5%)	35 (68.6%)	0.20
Female	18 (44.5%)	16 (31.4%)	
Anti-platelet therapy			
Absent	35 (87.5%)	43 (84.3%)	0.77
Present	5 (12.5%)	8 (15.7%)	
Tumor size			
Small (< 3 cm)	24 (60.0%)	31 (60.8%)	0.94
Large (≥ 3 cm)	16 (40.0%)	20 (39.2%)	
Tumor location			
Head	24 (60.0%)	32 (62.7%)	0.90
Body/Tail	16 (40.0%)	19 (37.3%)	
Histology^a^			
wel	23 (57.5%)	24 (47.1%)	0.40
Mod / por / muc/ anaplastic	17 (42.5%)	27 (52.9%)	
T category^b^			
T1/T2	3 (7.5%)	4 (7.8%)	0.95
T3	37 (92.5%)	47 (92.2%)	
Lymph node metastasis			
Absent	20 (50.0%)	17 (33.3%)	0.13
Present	20 (50.0%)	34 (66.7%)	
Lymphatic invasion^c^			
ly 0/1	30 (75.0%)	23 (45.1%)	0.005
ly 2/3	10 (25.0%)	28 (54.9%)	
Vascular invasion^d^			
v 0/1	14 (35.0%)	32 (62.7%)	0.011
v 2/3	26 (65.0%)	19 (37.3%)	
Intrapancreatic nerve invasion^e^			
ne 0/1	10 (25.0%)	11 (21.6%)	0.80
ne 2/3	30 (75.0%)	40 (78.4%)	
Surgical procedure			
PD	24 (60.0%)	34 (66.7%)	0.52
DP/TP	16 (40.0%)	17 (33.3%)	
Residual tumor			
R0	36 (90.0%)	47 (92.2%)	0.73
R1	4 (10.0%)	4 (7.8%)	
Neoadjuvant chemotherapy			
Absent	35 (87.5%)	43 (84.3%)	0.77
Present	5 (12.5%)	8 (15.7%)	
Adjuvant chemotherapy			
Absent	18 (45.0%)	21 (41.2%)	0.83
Present	22 (55.0%)	30 (58.8%)	

*PD* pancreatoduodenectomy, *DP* distal pancreatectomy, *TP* total pancreatectomy, *R0* no residual tumor, *R1* microscopic residual tumor

^a^Histology: wel, well differentiated type; mod, moderately differentiated type; por, tpoorly differentiated type; muc, mucinous carcinoma; anaplastic, anaplastic carcinoma

^b^T category: T1, tumor limited to the pancreas, ≤ 20 mm in the greatest dimension; T2, tumor limited to the pancreas, > 20 mm in greatest dimension; T3, tumor extends beyond the pancreas, but without involvement of the celiac artery or superior mesenteric artery

^c^ly 0–3: grade of lymphatic invasion

^d^v 0–3: grade of venous invasion

^e^ne 0–3: grade of perineural invasion

**Fig. 2:**
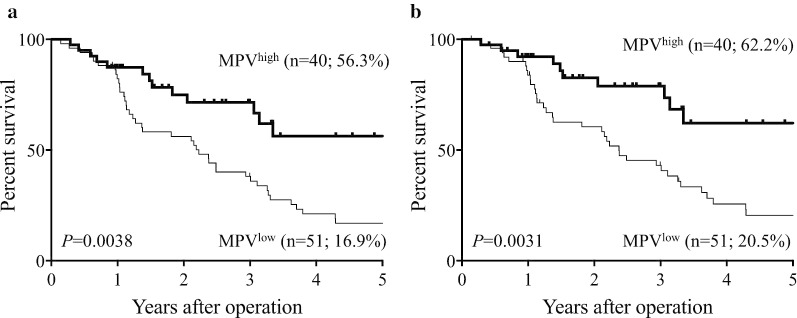
Survival curves according to MPV. **a** Overall survival. **b** Disease-specific survival

We then determined the prognostic significance of MPV. The 5-year OS rate was significantly lower in the MPV^low^ compared with the MPV^high^ group (16.9% vs. 56.3%; *P* = 0.0038; Fig. 2a), while the 5-year DSS rates were 20.5% and 62.2% in the MPV^low^ and MPV^high^ groups, respectively (*P* = 0.0031; Fig. 2b). Multivariate analysis identified MPV as an independent prognostic indicator of OS, together with lymph node metastasis, lymphatic invasion, residual tumor, adjuvant chemotherapy, and CA19-9 (Table 2). Furthermore, MPV was an independent prognostic indicator of DSS, together with lymph node metastasis, vascular invasion, residual tumor, adjuvant chemotherapy, and CA19-9 (Table 2).

**Table 2:** Multivariate analyses of clinicopathological factors of patients with pancreatic cancer

		Overall survival			Disease specific survival		
		HR	95%CI	P value	HR	95%CI	P value
Age	≥ 75 vs. < 75 Years	1.518	0.665–3.466	0.32	1.607	0.646–3.996	0.31
Sex	Male vs. female	0.653	0.301–1.413	0.28	0.682	0.294–1.583	0.37
Histology	Well vs. others	0.890	0.500–1.584	0.69	0.876	0.465–1.649	0.68
Tumor location	Head vs. others	1.089	0.096–12.423	0.95	1.784	0.145–21.936	0.65
Operative procedure	PD vs. DP/TP	1.419	0.136–14.841	0.77	1.263	0.112–14.267	0.85
Tumor size	≥ 3 cm vs. < 3 cm	1.741	0.885–3.426	0.11	1.947	0.928–4.084	0.078
T category	T3 vs. T1/2	1.385	0.406–4.170	0.56	0.902	0.288–2.824	0.86
Lymph node metastasis	Present vs. absent	3.261	1.492–7.127	0.003	2.721	1.173–6.308	0.020
Lymphatic invasion	ly2/3 vs. ly0/1	1.975	1.009–3.868	0.047	1.749	0.837–3.651	0.14
Vascular invasion	v2/3 vs. v0/1	1.913	0.930–3.934	0.078	2.796	1.253–6.308	0.012
Intrapancreatic nerve invasion	ne2/3 vs. ne0/1	1.506	0.700–3.237	0.30	1.592	0.694–3.650	0.27
Residual tumor	R1 vs. R0	5.165	1.722–15.493	0.003	8.496	2.620–27.549	< 0.001
Neoadjuvant chemotherapy	Present vs. absent	0.559	0.230–1.358	0.20	0.500	0.188–1.331	0.165
Adjuvant chemotherapy	Present vs. absent	0.346	0.153–0.783	0.011	0.318	0.129–0.783	0.013
CA19-9	≥ 66.3 vs. < 66.3 U/ml	2.452	1.159–5.187	0.019	1.928	1.061–4.319	0.044
MPV	< 8.65 vs. ≥ 8.65 fL	2.247	1.007–5.012	0.048	3.578	1.411–9.074	0.007

See Table 1 for the details of histology, surgical procedure, T category, lymphatic invasion, vascular invasion, intrapancreatic nerve invasion, and residual tumor

*CI* confidence interval, *HR* hazard ratio, *CA19-9* carbohydrate antigen 19-9, *MPV* mean platelet volume

**Fig. 3:**
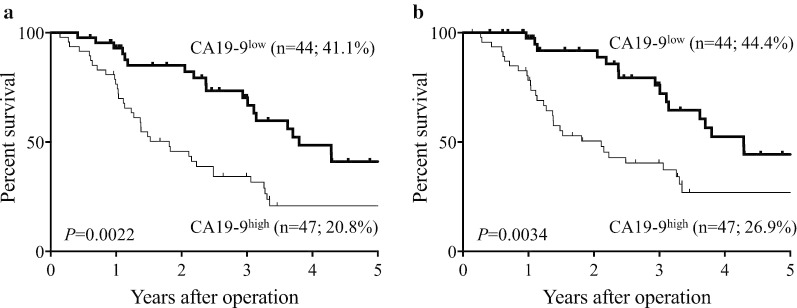
Survival curves according to serum CA19-9 level. **a** Overall survival. **b** Disease-specific survival

We next divided the patients into CA19-9^high^ (≥ 66.3; n = 47) and CA19-9^low^ (< 66.3; n = 44) groups according to the optimal cut-off value determined by ROC curve analysis. The prognosis of patients in the CA19-9^high^ group was significantly poorer than that of the CA19-9^low^ group in terms of both OS (5-year OS rates, 20.8% vs. 41.1%, respectively; *P* = 0.0022; Fig. 3a) and DSS (5-year DSS rates, 26.9% and 44.4%, respectively; *P* = 0.0034; Fig. 3b).

**Fig. 4:**
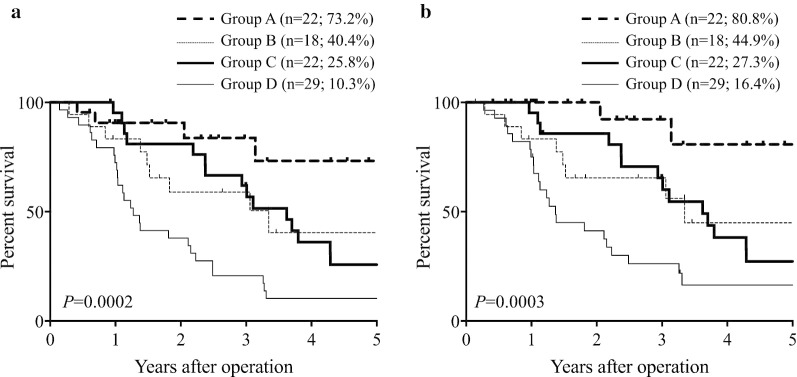
Survival curves according to the combined analysis of MPV and serum CA19-9 level. **a** Overall survival. **b** Disease-specific survival

**Fig. 5:**
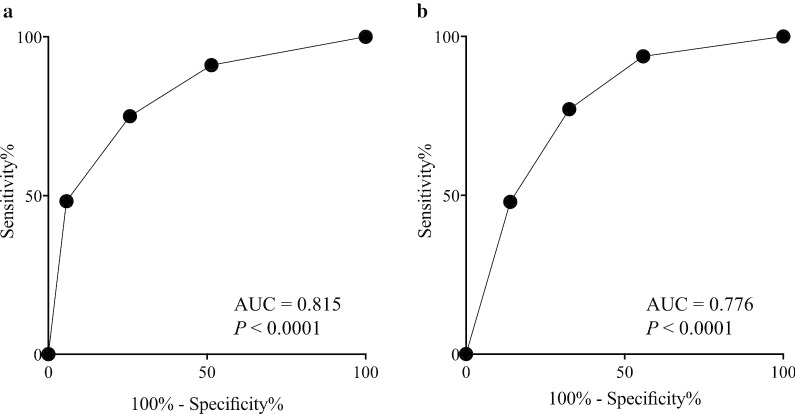
Receiver operating characteristic curves of the combined analysis of MPV and serum CA19-9 level. **a** Overall survival. **b** Disease-specific survival

We then determined if the patients’ prognosis could be predicted in more detail using MPV combined with CA19-9, as the most common oncological serum marker in PC patients. The relationship between MPV and serum CA19-9 level was not significant (r =  − 0.033; *P* = 0.755), and we therefore divided the patients into groups A (MPV^high^ and CA19-9^low^), B (MPV^high^ and CA19-9^high^), C (MPV^low^ and CA19-9^low^), and D (MPV^low^ and CA19-9^high^). The 5-year OS rates in the four groups were 73.2%, 40.4%, 25.8%, and 10.3%, respectively (*P* = 0.0002; Fig. 4a), and the 5-year DSS rates were 80.8%, 44.9%, 27.3%, and 16.4%, respectively (*P* = 0.0003; Fig. 4b). Finally, groups A, B, C, and D were assigned 0, 1, 2, and 3, respectively. ROC analysis showed that the AUC of the combined analysis of MPV and serum CA19-9 level was higher than that of either MPV or serum CA19-9 level alone for both OS (AUC = 0.815; *P* < 0.0001; Fig. 5a) and DSS (AUC = 0.776; *P* < 0.0001; Fig. 5b).

## Discussion

In this study, we determined the prognostic significance of platelet indices and showed a close relationship between decreased preoperative MPV and poor prognosis in patients with resected PC. Accumulating evidence indicates that platelets play important roles in tumor growth and metastasis. Platelets enhance the penetration of cancer cells through the blood vessel endothelial cell barrier, and prevent their clearance from the circulatory system by the immune cells [16]. Platelets also produce cytokines such as thymidine phosphorylase/platelet-derived endothelial cell growth factor (PDGF) and transforming growth factor-β1 (TGF-β1). PDGF is a well-described angiogenic factor capable of inducing microvessels at tumor sites, which is closely related to tumor growth and metastasis. Furthermore, mutant p53-induced upregulation of PDGF receptor beta drives invasion and metastasis in PC [17]. PDGF also inhibited the cell-killing effects of natural killer cells [18]. TGF-β1 is an immunosuppressive cytokine and also an angiogenic factor. Moreover, direct platelet–tumor cell contacts and platelet-derived TGF-β1 synergize to promote an epithelial–mesenchymal transition-like phenotype in vivo, which was found to enhance the migration and invasion of cancer cells [19]. These observations suggest the possible usefulness of platelet indices as a prognostic indicator in cancer patients, including patients with PC.

Thrombocytosis is often observed in cancer patients, given that many types of cancer cells and tumor-infiltrating cells stimulate platelet activation [20, 21]. Furthermore, thrombocytosis is related to a poor prognosis in various cancers [22–24]. However, the prognostic significance of platelet count in the current study was low, indicating the possibility that increased platelet count does not always reflect an increased number of activated platelets. Nonetheless, there was a close correlation between decreased MPV and poor prognosis in this study. Inflammation leads to the accumulation of large platelets at the site of inflammation, resulting in a decreased MPV in the peripheral blood [25]. Furthermore, excessive pro-inflammatory cytokines interfere with megakaryopoiesis, leading to an increased production of small-sized platelets from the bone marrow [26]. Gasparyan et al. showed that decreased MPV was related to the severity of inflammatory diseases, and that anti-inflammatory therapy increased MPV [27]. These results indicate that decreased MPV reflects the activation of platelets and the spread of inflammation in PC patients. It is likely platelet activation enhances the pro-tumorigenic effects of platelets, which reportedly worsen the prognosis of cancer patients. Because platelets play important roles as inflammatory regulators, their activation also enhances cancer-associated inflammation. Inflammation is closely related to cancer development and progression [28]. Platelet activation and the spread of inflammation might thus explain why a decreased MPV was closely related to poor prognosis in patients with PC. However, the current study did not provide data to directly support these hypotheses, and further investigations are required to clarify the detailed mechanisms responsible for the close relationship between decreased MPV and poor prognosis in PC.

We demonstrated that lymph node metastasis, residual tumor, adjuvant chemotherapy, and serum CA19-9 level were also independent prognostic indicators in this study. These are tumor-related indicators, whereas MPV is a patient-related indicator. Furthermore, our results found no significant correlation between MPV and these tumor markers, indicating the usefulness of MPV as a prognostic indicator regardless of tumor-related prognostic indicators. The difference in origin and lack of a significant correlation between MPV and tumor-related prognostic indicators encouraged us to determine the prognostic significance of the combination of MPV and tumor-related prognostic indicators. Among the tumor-related prognostic indicators, we focused on serum CA19-9, because serum CA19-9 levels can be easily obtained from preoperative blood examination. We demonstrated the prognostic superiority of the combined analysis of MPV and serum CA19-9 level compared with either MPV or serum CA19-9 level alone.

The present study had some limitations. First, its retrospective design was associated with some bias. Second, the number of patients was relatively small, and a larger trial is required to confirm our results. Third, the detailed mechanisms related to the poor prognosis in patients with MPV^low^ remain unclear, and further investigations are urgently required to elucidate these.

In conclusion, our study indicated the usefulness of preoperative MPV as a prognostic indicator in patients with resected PC. Because the detection of these serum markers is quick, convenient, and less-invasive, the combined analysis of MPV and serum CA19-9 level could be used as a prognostic indicator for PC patients in the routine clinical setting.

## Data Availability

The datasets used and/or analyzed during the current study are available from the corresponding author on reasonable request.

## Abbreviations

AUC: Area under curve
CA19-9: Carbohydrate antigen 19-9
CBC: Complete blood count
DSS: Disease-specific survival
MPV: Mean platelet volume
OS: Overall survival
PC: Pancreatic cancer
PDGF: Platelet-derived endothelial cell growth factor
PDW: Platelet distribution width
ROC: Receiver operating characteristic
TGF-β1: Transforming growth factor-β1
TNM: Tumor, node, metastasis
UICC: Union for International Cancer Control
